# Modeling maternal mortality in Bangladesh: the role of misoprostol in postpartum hemorrhage prevention

**DOI:** 10.1186/1471-2393-14-78

**Published:** 2014-02-20

**Authors:** Ndola Prata, Suzanne Bell, Md Abdul Quaiyum

**Affiliations:** 1 Bixby Center for Population, Health and Sustainability, School of Public Health, University of California at Berkeley, 229 University Hall, UC Berkeley, Berkeley, CA 94720-7360, USA; 2 Bixby Center for Population, Health and Sustainability, School of Public Health, University of California at Berkeley, 17 University Hall, UC Berkeley, Berkeley, CA 94720-7360, USA; 3 icddr,b, Centre for Reproductive Health, GPO Box 128, Dhaka 1000, Bangladesh

**Keywords:** Traditional birth attendant, Bangladesh, Postpartum hemorrhage, Maternal mortality, Misoprostol, Delivery mat, Monte Carlo

## Abstract

**Background:**

Bangladesh is one of the few countries that may actually achieve the fifth Millennium Development Goal (MDG) in time, despite skilled birth attendance remaining low. The purpose of this paper is to examine the potential role misoprostol can play in the decline of maternal deaths attributed to postpartum hemorrhage (PPH) in Bangladesh.

**Methods:**

Using data from a misoprostol and blood loss measurement tool feasibility study in Bangladesh, observed cause specific maternal mortality ratios (MMRs) were estimated and contrasted with expected ratios using estimates from the Bangladesh Maternal Mortality Survey (BMMS) data. Using Crystal Ball 7 we employ Monte Carlo simulation techniques to estimate maternal deaths in four scenarios, each with different levels of misoprostol coverage. These scenarios include project level misoprostol coverage (69%), no (0%), low (40%), and high (80%) misoprostol coverage. Data on receipt of clean delivery kit, use of misoprostol, experience of PPH, and cause of death were used in model assumptions.

**Results:**

Using project level misoprostol coverage (69%), the mean number of PPH deaths expected was 40 (standard deviation = 8.01) per 100,000 live births. Assuming no misoprostol coverage (0%), the mean number of PPH deaths expected was 51 (standard deviation = 9.30) per 100,000 live births. For low misoprostol coverage (40%), the mean number of PPH deaths expected was 45 (standard deviation = 8.26) per 100,000 live births, and for high misoprostol coverage (80%), the mean number of PPH deaths expected was 38 (standard deviation = 7.04) per 100,000 live births.

**Conclusion:**

This theoretical exercise hypothesizes that prophylactic use of misoprostol at home births may contribute to a reduction in the risk of death due to PPH, in addition to reducing the incidence of PPH. If findings from this modeling exercise are accurate and uterotonics can prevent maternal death, misoprostol could be the tool countries need to further reduce maternal mortality at home births.

## Background

Maternal mortality remains a major challenge in many settings with more than 99% of global maternal deaths still occurring in low-resource countries [1,2]. The lifetime risk of a woman dying in pregnancy or within 42 days of its termination is approximately 1 in 150 in developing countries and 1 in 38,000 in developed countries [3]. The fifth Millennium Development Goal (MDG) strove to address the high levels of maternal mortality throughout the world by calling for a reduction in the maternal mortality ratio (MMR) by three-quarters from 1990 to 2015 [4]. The international maternal health community’s strategy to achieve this goal has primarily focused on increasing access to skilled birth attendants (SBAs) and emergency obstetric care (EmOC), but women delivering outside of health facilities have largely not benefited from these efforts [5].

Nearly half of all births worldwide (46%) still occur outside of an institutional setting, attended by a traditional birth attendant (TBA), a relative, or no one [6,7]. Part of the reason that more deliveries are not attended by an SBA is the fact that many settings with high MMRs have low ratios of SBAs to women and progress in this regard has remained slow [8]. TBAs that attend these births typically do not have appropriate technologies to manage or treat complications, which explains many studies’ findings that TBA trainings are not effective at improving maternal survival [9]. Despite these discouraging findings, research has shown that approximately 80% of maternal deaths are due to avoidable causes, among which hemorrhage is a leading cause [10,11]. Hemorrhage is estimated to be responsible for approximately 35% of maternal deaths in developing countries, although this varies widely across regions/countries [12,13]. Postpartum hemorrhage (PPH), blood loss of 500 ml or more, is responsible for the majority of the maternal deaths due to hemorrhage [14]. PPH poses a significant public health challenge in low-resource settings because of its low predictability [15] and the speed at which it kills; without intervention, 88% of women who die of postpartum hemorrhage die within four hours of delivery [16].

Misoprostol is a generic, low cost, heat-stable uterotonic that can be orally administered in tablet form for the prevention of PPH. Operations research has demonstrated the safety, feasibility, and acceptability of low-level providers, or women themselves, administering misoprostol for PPH prevention during home deliveries in low-resource settings [17-23]. In addition, two randomized controlled trials (RCTs) evaluated the use of 600 micrograms of misoprostol compared to a placebo to prevent PPH in home births [24,25]. In rural India, Derman *et al.* demonstrated a statistically significant 47% reduction in the relative risk of PPH (RR = 0.53, 95% CI 0.39-0.74) [24]. Mobeen *et al.* illustrated that TBAs using misoprostol at home births in Pakistan could reduce the relative risk of PPH by 24% (RR = 0.76, 95% CI 0.59-0.97) [25]. Based on a review of existing evidence, the World Health Organization (WHO) added misoprostol to the Essential Medicines List for the prevention of PPH in 2011 and now supports non-skilled birth attendant administration of misoprostol at home deliveries [26,27]. Incorporating misoprostol at home deliveries has the potential to avert PPH attributable maternal deaths at the community level where most women in low-resource settings give birth.

Bangladesh is one of the few countries that may actually achieve the fifth MDG in time, despite SBA attendance remaining low. Recent estimates put the MMR at 194 maternal deaths per 100,000 live births, which is impressive given that approximately 71% of births take place at home, of which only 4.3% are attended by a skilled provider [28]. Estimates show that 31% of maternal mortality is attributable to hemorrhage in this context, highlighting the need for community-based interventions like misoprostol to reduce preventable maternal deaths [29].

The purpose of this paper is to examine the potential role misoprostol can play in the decline of maternal deaths attributed to PPH at home births in Bangladesh. This study uses empirical data from a PPH prevention program and findings from previously published RCTs to model the number of PPH deaths that would result from four different scenarios of misoprostol coverage. Scenario 1 represents the project level coverage of 69%; scenario 2, 3, and 4 hypothetically represent no coverage (0%), low coverage (40%), and high coverage (80%), respectively.

### Study context

Operations research to evaluate the feasibility and acceptability of scaling-up community-based use of misoprostol and a postpartum blood loss measurement tool was conducted in six districts of the Rangpur Division in Bangladesh from May 2009 through September 2010. The study was a collaboration between a local non-governmental organization, Rangpur Dinajpur Rural Services (RDRS), the International Centre for Diarrheal Disease Research, Bangladesh (icddr,b) in Dhaka, the University of California, Berkeley Bixby Center for Population, Health, and Sustainability, and Venture Strategies Innovations. A total of 696 RDRS field staff, including 585 RDRS TBAs, received a two day training on misoprostol (function, dosage, timing of administration, side effects and their management, etc.) and use of the blood measurement tool, which was designed to absorb 500 mL of blood after delivery of the baby and be a visual cue to indicate the onset of PPH when the mat was full. The training also covered identifying high-risk pregnancies, danger signs in pregnancy, referral procedures, stages of labor, newborn resuscitation, maternal infection, and use of the clean delivery kits. The misoprostol and blood measurement tool were added to RDRS’s existing clean delivery kit (CDK) at no extra charge and were distributed to clients registered with RDRS during antenatal care (ANC), or at the time of delivery by a TBA.

A total of 118,500 women enrolled in the study, of which 77,337 delivered during the project monitoring period. Of those, 67,611 (87%) delivered at home. Verbal autopsies for all maternal deaths that occurred in the study areas during the project period were conducted. Ethical permission for the study was granted by the University of California, Berkeley (CPHS # 2010-01-619). Additional information on the study can be found in the final report [30]. Study results on training TBAs to use misoprostol and the blood loss measurement tool in home births in Bangladesh have been published elsewhere [31].

## Methods

Cause specific maternal mortality data used in this analysis came from verbal autopsies conducted in the project described above. Verbal autopsy interviews were conducted with persons who were most familiar with the specifics surrounding the maternal death using an adapted version of the tool available from the WHO [for more recent version, see: [32]]. Each verbal autopsy questionnaire was independently reviewed by two physicians in order to determine an immediate cause of maternal death using the International Classification of Disease (ICD) 10 [33]. Final cause of death categories used include the main obstetric or direct causes of maternal death and a group of indirect causes. Cause of death categories used in this study differ from those in the study’s final report because researchers combined cause of death categories to better reflect the Bangladesh Maternal Mortality Survey (BMMS) categorization [29,30]. Observed cause specific maternal mortality ratios are estimated and contrasted with expected ratios using estimates from the BMMS data [29].

**Figure 1:**
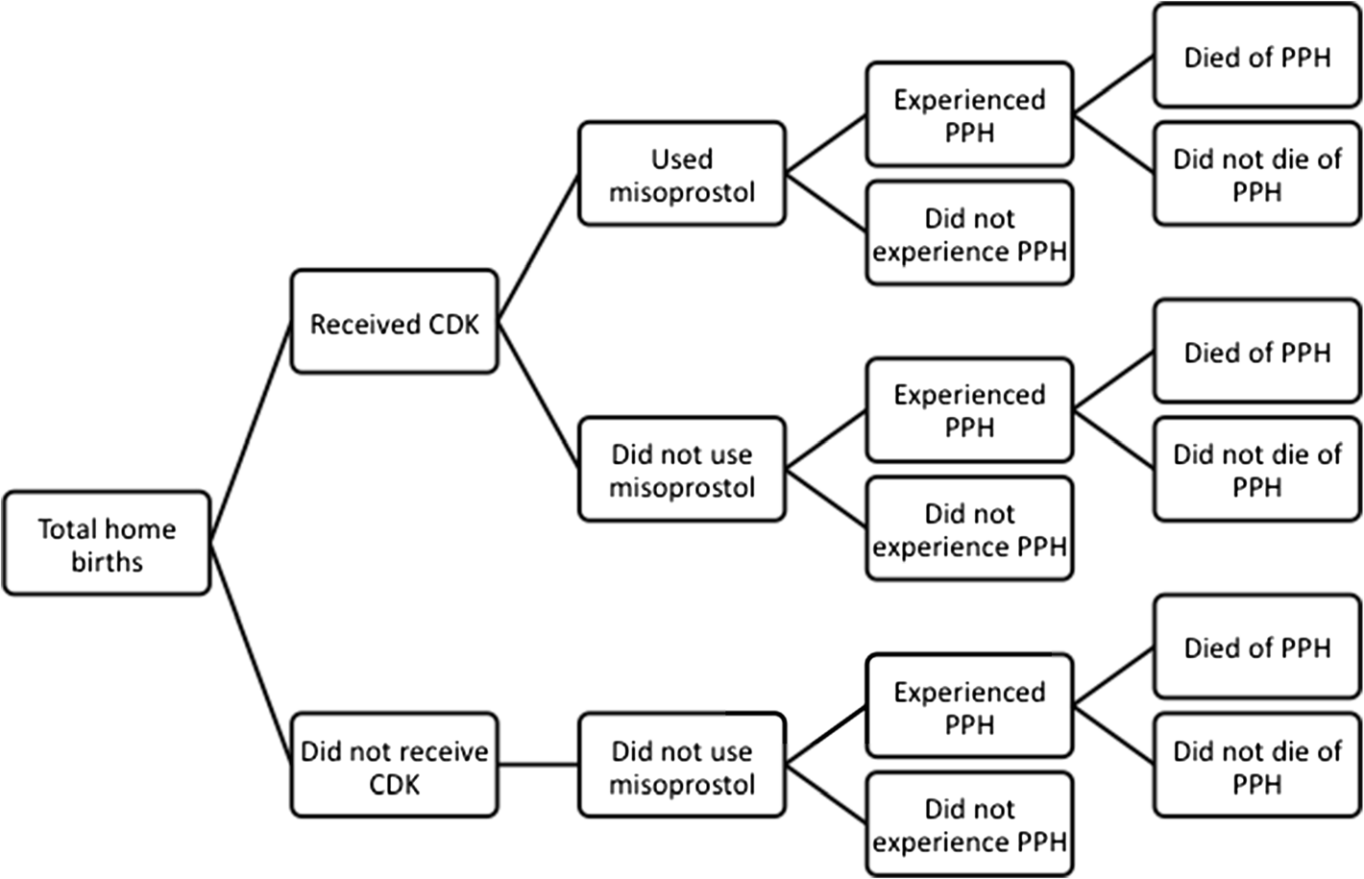
Flow chart of categorization of women who delivered at home by receipt of CDK, use of misoprostol, experience of PPH, and death due to PPH.

For model building, we use project data on CDK coverage and misoprostol utilization from ANC cards and cause of death data from the verbal autopsies to categorize women (Figure 1). Coverage data includes the percentage of women who received the CDK, and misoprostol utilization is the percent of those who received the CDK who used the misoprostol (Table 1). To estimate the effectiveness of misoprostol in reducing PPH we use data from two RCTs where misoprostol was shown to reduce PPH by 24% to 47% when administered at home deliveries by lay providers (Mobeen, RR = 0.76, 95% CI 0.59-0.97; Derman, RR = 0.53, 95% CI 0.39-0.74) [24,25]. In these RCTs, 17% and 6% of women experienced PPH if they took misoprostol, compared to 22% and 12% if they did not [24,25]. Reviews of RCTs comparing misoprostol to a placebo, although not necessarily at the community level or involving lay healthcare workers, have found risk ratios within this range, but not all findings have been significant [34-36]. The intervention did incorporate an absorbent delivery mat to visually estimate blood loss, but the delivery mat was not always utilized (68% of home births used the mat), nor were data collected on whether the mat was filled. Women self-reported whether they thought they experienced PPH at a postnatal appointment. If they used the mat, PPH was reported based on visual estimation resulting from the mat being soiled. However, in the case of a maternal death, someone involved in the delivery reported whether he/she thought the woman experienced PPH. We believe that blood loss estimation to establish PPH was more accurately measured in the RCTs, thus we used the prevalence of PPH among those who did and did not take misoprostol from previous RCTs in our modeling assumptions. Mortality data according to whether or not the woman had used misoprostol and died of PPH was taken from the verbal autopsy reports from the project data (Table 1).

**Table 1:** Assumptions in Monte Carlo modeling scenarios

	**%**				
	**Minimum**	**Likeliest**	**Maximum**	**Distribution**	**Source of assumption**
**Assumptions used in all scenarios**					
% used misoprostol had PPH	6	12	17	Triangle	RCTs
% used misoprostol died of PPH	0.2	0.3	0.4	Triangle	Project data
% did not use misoprostol had PPH	12	17	22	Triangle	RCTs
% did not use misodied of PPH	0.2	0.3	0.4	Triangle	Project data
**Scenario specific assumptions**					
Scenario 1: Project data					
% received CDKs	49	69	89	Triangle	Project data
% received CDK used miso	80	94	100	Triangle	Project data
% did not use miso	0	6	20	Triangle	Project data
Scenario 2: No misoprostol coverage					
% received CDKs	0	0	0	Uniform	Set level
% received CDK used miso	0	0	0	Uniform	Set level
% did not use miso	100	100	100	Uniform	Set level
Scenario 3: Low misoprostol coverage					
% received CDKs	20	40	60	Triangle	Set level
% received CDK used miso	80	94	100	Triangle	Project data
% did not use miso	0	6	20	Triangle	Project data
Scenario 4: High misoprostol coverage					
% received CDKs	60	80	100	Triangle	Set level
% received CDK used miso	80	94	100	Triangle	Project data
% did not use miso	0	6	20	Triangle	Project data

We employed Monte Carlo modeling techniques using Crystal Ball 7, a stochastic modeling supplement for Excel [37]. Monte Carlo simulations are computational algorithms that utilize repeated random sampling by running simulations many times as a way of estimating probabilities. Assumption parameters are set for all variables included in the simulation, which determine the likely range of possibilities. Data on receipt of CDK, use of misoprostol, experience of PPH, and cause of death were used to estimate parameters for the model. Most assumptions were assigned a triangle distribution, where the likeliest value in the distribution was drawn from the empirical evidence in our study or the literature, with an associated minimum and maximum value (Table 1).

Our modeling began with a hypothetical population of 100,000 singleton live births from Bangladeshi women, all of whom delivered at home. As seen in Figure 1, this population was divided into those who received a CDK and those who did not, and then those who used misoprostol and those who did not. We divided these groups into those who experienced PPH and those who did not. Lastly, we divided those who experienced PPH into those who died from PPH and those who did not. Thus our main outcome in the model was the total number of PPH related deaths. The percentages in Table 1 were used in the Monte Carlo simulation to calculate the likely probability that a woman would be in each of these categories along the “decision tree” presented in Figure 1, and ultimately, the range of probabilities that she would die of postpartum hemorrhage.

We influenced the coverage of misoprostol to create a series of scenarios, each of which was run 50,000 times. In scenario 1, the coverage assumption was set using the data from the study (69%); in scenario 2 we set the misoprostol coverage at 0%; in scenario 3 we set the misoprostol coverage low at 40%; and in scenario 4 we set the misoprostol coverage high at 80%. We also calculated overall MMRs and the associated number of maternal deaths country-wide for each scenario, keeping all other cause specific maternal mortality ratios the same using BMMS data. We wanted to generate overall MMR estimates that incorporated scenario specific PPH MMRs and were generalizable to the entire country but there was evidence of significant differences between project and BMMS cause specific MMRs, thus we used BMMS cause specific MMRs. We used the most recent Bangladesh estimate for the total number of annual births to calculate the total number of maternal deaths in each of the four scenarios [38]. Lastly, we conducted a perturbation of estimates for each scenario to investigate the robustness of the estimates to changes in underlying assumptions.

## Results

Overall, 117 women died during the study period, 54 (46%) of whom died of PPH or bleeding related causes (Table 2). Among women who delivered at home during the study period (67,611), 69% received the CDK; among those, 94% used the misoprostol. Among this sub-population of home deliveries that was used to inform the Monte Carlo simulation assumptions, 34 women died of PPH; 17 who took misoprostol, 16 who did not, and 1 whose misoprostol usage is unknown (Table 2). Table 2 provides additional information on the cause of death, timing of death, place of delivery, and attendant at delivery among all maternal deaths in the study.

**Table 2:** Attributed cause of death, timing of death, use of misoprostol, place of delivery, and provider at delivery from verbal autopsy data from operations research in Bangladesh assessing the feasibility and acceptability of community-based use of misoprostol and a postpartum hemorrhage blood loss measurement tool (N=117)

**Cause of death**	**Number of maternal deaths**	**% distribution by cause**	**Timing of death**^**1**^			**Took misoprostol**	**Place of delivery**^**2**^		**Attendant at delivery**	
			**Before delivery**	**During delivery**	**After delivery**		**Home**	**Facility**	**Skilled attendant**^**3**^	**Unskilled attendant**^**4**^
**Direct obstetric causes**	**110**	**94.0**	**24**	**17**	**67**	**27**	**44**	**34**	**37**	**67**
PPH and bleeding related	54	46.2	4	7	42	17	34	15	19	31
Antepartum hemorrhage (APH)	5	4.3	1	3	1	1	0	2	1	4
Eclampsia	27	23.1	11	3	13	3	4	12	11	14
Obstructed labor	2	1.7	0	2	0	0	0	0	0	2
Ruptured uterus	2	1.7	1	0	1	1	1	0	0	2
Other direct causes	20	17.1	7	2	10	5	5	5	6	14
**Indirect causes**	7	6.0	3	0	4	0	3	1	1	6
**Total**	**117**	**100.0**	**27**	**17**	**71**	**27**	**47**	**35**	**38**	**73**

^1^Two women are missing information on timing of death, one who died of PPH and onewho died of other direct causes.

^2^Options were: home, upazilla health complex, upazilla health and family welfare center, private hospital/clinic, government hospital, maternal and child welfare center, in between hospital and home, other, and not applicable. “Facility” is all categories except home, between hospital and home, other, and not applicable.

^3^Skilled attendant is doctor or nurse/midwife.

^4^Unskilled includes RDRS TBA, untrained TBA, relative, village doctor, self, and other.

Based on the number of maternal deaths, we calculated overall and cause specific MMRs for the project and compared these to the expected MMRs based on the BMMS data (Table 3). The project estimated PPH specific MMR was 71.9 deaths per 100,000 live births whereas the BMMS PPH [combined with antepartum hemorrhage (APH)] cause specific MMR was 60.2 deaths per 100,000 live births. This difference was not statistically significant, though some of the cause specific MMRs were statistically significantly different. These include obstructed labor, other direct causes, and indirect causes. These differences are likely due to differences in the categorization of maternal deaths. We were unable to compare cause specific MMRs for APH and ruptured uterus because these cause of death categories were not used in the BMMS report.

**Table 3:** Observed and expected cause specific maternal mortality ratios

**Cause of death**	**Number of maternal deaths**	**Deaths/100,000 live births**			**p-value**
		**Project estimated MMR**	**Expected MMR (based on BMMS)**^**1**^	**Difference**	
PPH and bleeding related^2^	54	71.9	60.2	11.73	0.309
Antepartum hemorrhage (APH)	5	6.7	N/A	N/A	N/A
Eclampsia	27	36.0	38.6	-2.63	0.763
Obstructed labor	2	2.7	12.5	-9.84	0.012
Ruptured uterus	2	2.7	N/A	N/A	N/A
Other direct causes	20	26.6	11.4	15.2	0.014
Indirect causes	7	9.3	68.2	-58.9	<0.001
Total	117	156	194	-38	0.042

^1^Also had category “undetermined”, MMR=2.3.

^2^Hemorrhage (PPH and APH) for BMMS data.

Table 4 contains results from the four scenarios modeled using Monte Carlo simulation. We used project level data along with results from RCTs investigating the efficacy of misoprostol for PPH prevention at home births to inform the assumptions for the model. Using project level misoprostol coverage (69%), the mean number of PPH deaths expected was 40 (standard deviation = 8.01) per 100,000 live births. Assuming no misoprostol coverage (0%), the mean number of PPH deaths expected was 51 (standard deviation = 9.30) per 100,000 live births. For low misoprostol coverage (40%), the mean number of PPH deaths expected was 45 (standard deviation = 8.26) per 100,000 live births, and for high misoprostol coverage (80%), the mean number of PPH deaths expected was 38 (standard deviation = 7.04) per 100,000 live births.

**Table 4:** Mean number of PPH specific maternal deaths and associated PPH specific and overall MMRs in a hypothetical population of 100,000 Bangladeshi women who delivered live births at home across 4 scenarios modeled using Crystal Ball

**Scenario**	**Misoprostol coverage**	**Mean number of PPH deaths/PPH specific MMR**	**Range of number of PPH deaths**	**SD of the sample mean**	**Expected number of all other maternal deaths**^**1**^	**Overall MMR**^**1**^	**Estimated number of maternal deaths**^**2**^
Project level	69%	40	15-76	8.01	134	174	5,286
No misoprostol	0%	51	25-85	9.30	134	185	5,620
Low misoprostol	40%	45	20-83	8.26	134	179	5,438
High misoprostol	80%	38	17-70	7.04	134	172	5,225

^1^Calculated from BMMS MMRs (overall MMR, 194; overall MMR excluding PPH specific MMR, 134) with the assumption that all other causes of death happened at the same level as BMMS.

^2^Based on recent estimate of 3,038,000 annual births [38].

Using BMMS data for all cause specific MMRs other than PPH (which total 134 per 100,000 live births) and holding these ratios constant regardless of the level of misoprostol coverage, we calculated overall MMRs for each scenario and applied these MMRs to Bangladesh’s most recent estimate for the total number of annual births [38]. This allowed us to calculate the total estimated number of maternal deaths for the country (Table 4). Using the project level scenario, 5,286 maternal deaths would occur at the national level. For the no misoprostol, low misoprostol, and high misoprostol scenarios, 5,620, 5,438, and 5,225 maternal deaths would occur, respectively, at the national level. This indicates that, given the assumptions of the scenarios, an estimated 395 PPH related maternal deaths could be averted by increasing the misoprostol coverage at home births from 0% to 80% nationwide.

**Figure 2:**
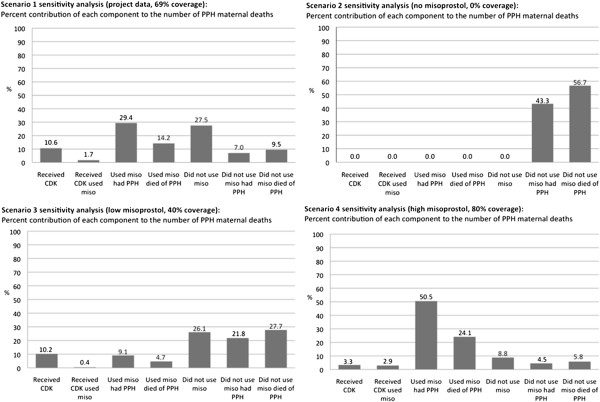
Results from sensitivity analyses.

The results from the sensitivity analyses reveal varying levels of contribution of each component to the variance in the number of PPH maternal deaths based on the assumptions in each scenario (Figure 2). In scenario 1 (project data, 69% coverage), “using misoprostol and having PPH” and “not using misoprostol” were the components that contributed the most to the variance in the number of PPH deaths, 29% and 28%, respectively. In scenario 2 (no misoprostol, 0% coverage), “not using misoprostol and dying of PPH” and “not using misoprostol and having PPH” were the only factors, the former of which contributed the greatest to the variance in the number of PPH maternal deaths; “not using misoprostol and dying of PPH” contributed slightly more than half of the variance in the total number of PPH deaths. In scenario 3 (low misoprostol, 40% coverage), “not using misoprostol and dying of PPH” had the highest level of contribution to the variance in the outcome (28%), but “not using misoprostol” (26%) and “not using misoprostol and experiencing PPH” (22%) were similarly high. In scenario 4 (high misoprostol, 80% coverage), “using misoprostol and experiencing PPH” was the component that contributed most to the variance in the number of PPH maternal deaths at 51%.

## Discussion

Results theorize the potential contribution misoprostol utilization at home births can have on the number of PPH attributable deaths and how this might affect the overall number of maternal deaths in Bangladesh if this intervention were brought to scale. This is the first modeling exercise that uses maternal mortality data from a single intervention to investigate the potential impact of prophylactic misoprostol use on PPH mortality. The data for this exercise come primarily from the largest misoprostol/PPH prevention intervention conducted in a low-resource setting, providing ample amounts of data from which we could formulate assumptions for the simulations.

Our findings estimate the range of PPH specific MMRs and total number of maternal deaths that could result from four different scenarios with varying levels of misoprostol coverage. If Bangladesh had no misoprostol coverage at home births, the estimated PPH specific MMR would be 51 deaths per 100,000 live births. This is in contrast to the estimated PPH specific MMR with high (80%) misoprostol coverage, which would result in 38 deaths per 100,000 live births. If brought to scale, based on the assumptions of the simulations, this intervention has the potential to greatly reduce the total number of maternal deaths. These findings highlight how a PPH prevention intervention at home births could have a substantial impact on maternal mortality, especially in countries such as Bangladesh where a large number of births take place at home.

Despite the vast amount of data and number of women involved in the study, this project and associated analyses had several limitations. Most importantly, alternative explanations could also be used to explain the difference in mortality among those who did and did not take misoprostol. Perhaps the difference is simply due to an ecological association, whereby women who live closer to a facility are more likely to access ANC services, receive and use the misoprostol, and then more likely to access and receive life-saving care in the event of complications. Previous research found that increased distance to a facility was associated with increased risk of maternal mortality, but this was among women who delivered with a skilled provider; this association did not hold among those who delivered without a skilled provider [39]. All the women in the data used to calculate the assumptions for this modeling exercise delivered at home without a skilled provider, they were all enrolled in private sector ANC through RDRS (who recruited women in these rural areas door-to-door), and they could all request an RDRS TBA to be present at their delivery, who brought CDKs in the event that the woman had not already received one. Thus we feel it is unlikely that women who did not receive/use misoprostol were systematically further from a health facility than those who did. However, we acknowledge it is possible that underlying factors inherent in the woman who used the misoprostol and the mat (e.g. motivation, knowledge, etc.) also made her more likely to be transferred earlier and to receive appropriate life-saving care since the women were not randomized to use the interventions or not. The strongest evidence against this explanation is that the probability of death among those who experienced PPH was equal among those who did and did not take misoprostol (0.3%), thus mortality was reduced via a decrease in the incidence of PPH, not a decrease in the risk of death once one experienced PPH.

On the whole, the PPH MMRs from the simulations are lower than that of the project data and the BMMS data. We hypothesize that it could be due to the fact that some women who were at high risk, who experienced obstetric complications early on during labor, or who had heavy bleeding immediately after labor could have been transferred to a facility and not been categorized as having delivered at home; per training instructions, immediate transfer was promoted in cases of heavy bleeding. This would likely result in an increase in the PPH MMR among facility deliveries and a decrease in the PPH MMR among home births than would otherwise have been expected.

The cause of death categories in the BMMS report do not align with our categories. For instance, the BMMS report combines PPH and APH deaths into “hemorrhage” whereas we wanted to isolate PPH attributable deaths, thus we kept PPH as a separate category. This difference, among others, resulted in the cause specific MMRs not being entirely comparable. Although not ideal, we include them nonetheless for exploration, knowing also that the BMMS MMR is for the entire country, accounting for rural and urban deliveries and home and facility deliveries; the project data contains 87% home deliveries, which is above the national average of 71%, and the entire population is rural [28]. One would expect the project estimate to be higher given the great percentage of home deliveries and rural residents in comparison to the country as a whole, but our overall MMR was in fact lower. This could be due to the fact that all women were enrolled with a local NGO that provided ANC and delivery services. Despite these differences, using the BMMS MMR as the expected MMR and contrasting that with scenario specific PPH MMRs allows us to make broad yet tenuous generalizations about the total number of maternal deaths expected at a national level. Because the scenario specific PPH MMRs were much lower than the study or the BMMS PPH MMRs, our findings are likely conservative estimates of the total number of PPH deaths that could be averted at the national level in Bangladesh.

The use of verbal autopsy to determine cause of death is a limitation. Because of the number of women involved in the study and the fact that nearly all deliveries were conducted at home without a skilled provider, researchers had to rely on interviews with someone (often a close relative) involved in the delivery of each woman who died in the study. Given the nature of the intervention, which included an IEC campaign about the dangers of PPH specifically, there was a heightened awareness among women and families of the risk of PPH, and a blood collection mat was used to assess bleeding. This could have contributed to increases in the reporting of bleeding when other symptoms or signs were ignored and not reported, resulting in differential misclassification of cause of death, with more deaths being classified by those interviewed for the verbal autopsy as a death due to excessive bleeding. This would help to explain why the study’s PPH specific MMR was much higher than the BMMS estimate.

The assumption that the rate of PPH mortality is constant across the population is another limitation. The more marginalized women who do not attend ANC and who do not receive a CDK likely have a higher PPH mortality rate than those who do. We were unable to estimate and incorporate these potential differences in our modeling, thus the findings do not accurately capture the fact that increasing coverage may not be linearly associated with reductions in PPH mortality.

Additionally, incomplete data on one PPH maternal death resulted in the exclusion of the case because we could not categorize the death according to misoprostol use. The potential difference that would result from the correct categorization of this woman would not be substantial, thus we do not feel this significantly biased our results.

Despite these limitations, the results of this modeling exercise hypothesize the potential differences in overall maternal deaths that could result from varying levels of misoprostol coverage throughout the country. Compared to no misoprostol coverage, findings indicate that achieving 80% coverage (with 94% utilization among that 80%) might be associated with a reduction in the overall MMR of 13 maternal deaths (185 to 172) per 100,000 live births; this is equivalent to a 7% reduction in overall maternal mortality. The 7% reduction represents about a quarter (23%) of the 31% of total maternal mortality that is attributable to PPH in Bangladesh. The remaining 77% of PPH maternal mortality could require a stronger uterotonic, more uterotonics, or other interventions in order to prevent mortality among these cases. Little is known about the causes of PPH that result in mortality, thus it could be that of the 31% of maternal deaths due to PPH in Bangladesh, only 23% of it can be prevented by misoprostol and the remainder would require other interventions; this is less than previously thought given the assumption that most PPH is due to uterine atony, which would be responsive to uterotonics.

The overall reduction in maternal mortality from this study is actually lower than that observed in other modeling exercises [40-42]. If true, this could mean a decrease in the cost-effectiveness of this approach [40,41]. Use of misoprostol for PPH treatment is a more cost-effective intervention [41], but it would require getting more women to deliver in facilities and encouraging early transfer among home births in order to treat PPH cases, which are difficult to predict [15]. Nonetheless, the hypothetical scenarios in this modeling exercise present the possibility that many lives could be saved in Bangladesh, and other countries, by increasing coverage of misoprostol availability among women delivering at home; a potential 7% reduction in MMR is not an insignificant number of lives saved. Additional empirical evidence is needed to confirm the results of this modeling exercise and the incidence of PPH mortality among women who do and do not take misoprostol.

## Conclusion

This theoretical exercise hypothesizes that prophylactic use of misoprostol at home births may contribute to a reduction in the risk of death due to PPH, in addition to reducing the incidence of PPH, if uterotonics can in fact prevent maternal death. Even after the WHO’s decision to support misoprostol use for PPH prevention at home births, there have been continued opponents of misoprostol use for this purpose, claiming insufficient evidence [43]. Other researchers pooled existing study data to determine the potential impact of misoprostol on PPH mortality and found that its use neither increased nor decreased mortality or severe morbidity [44]. The findings from our modeling exercise support the case for misoprostol use at home births in low-resource settings and hypothesize the potential reductions in maternal death due to PPH that can be achieved with various levels of misoprostol coverage, incorporating empirical data from a large field trial. If findings from this modeling exercise correspond to the possible effect of misoprostol use on PPH mortality, misoprostol could be the tool that countries need in order to further reduce maternal mortality at home births.

## Abbreviations

ANC: Antenatal care; BMMS: Bangladesh Maternal Mortality Survey; CDK: Clean delivery kit; EmOC: Emergency obstetric care; ICD: International Classification of Disease; icddr,b: International Centre for Diarrheal Disease Research, Bangladesh; MDG: Millennium Development Goal; MMR: Maternal mortality ratio; NGO: Non-governmental Organization; PPH: Postpartum hemorrhage; RDRS: Rangpur Dinajpur Rural Services; SBA: Skilled birth attendant; TBA: Traditional birth attendant; WHO: World Health Organization.

## Competing interests

The authors declare that they have no competing interests.

## Authors’ contributions

NP had the idea for this manuscript and helped with the analyses and the writing. SB conducted the analyses and did much of the writing. MAQ helped with the writing and oversaw data collection in-country. All authors read and approved the final manuscript.

## Pre-publication history

The pre-publication history for this paper can be accessed here:

http://www.biomedcentral.com/1471-2393/14/78/prepub
